# Effect of probiotics and related supplements on glycemic control in pediatric patients with type 1 diabetes mellitus: a systematic review and meta-analysis of clinical trials

**DOI:** 10.3389/fped.2025.1633694

**Published:** 2025-10-23

**Authors:** Hanyu Huang, Deyu Ma, Yan Zhou, Liping Wu

**Affiliations:** ^1^ Department of Nursing, Children’s Hospital of Chongqing Medical University, National Clinical Research Center for Child Health and Disorders, Ministry of Education Key Laboratory of Child Development and Disorders, Chongqing Key Laboratory of Pediatric Metabolism and Inflammatory Diseases, Chongqing, China; ^2^ School of Nursing, Chongqing Medical University, Chongqing, China; ^3^ Department of Radiology, Chongqing Health Center for Women and Children, Chongqing, China; ^4^ Department of Radiology, Women and Children’s Hospital of Chongqing Medical University, Chongqing, China; ^5^ Ministry of Education Key Laboratory of Clinical Laboratory Diagnostics, College of Laboratory Medicine Chongqing Medical University, Chongqing, China

**Keywords:** type 1 diabetes mellitus, children, probiotics, synbiotics, glycemic control, hemoglobin A1c, meta-analysis

## Abstract

**Objective:**

Type 1 diabetes mellitus (T1DM) is a common autoimmune disease in children, characterized by the destruction of pancreatic β-cells. Despite treatment advancements, many patients struggle with glycemic control. Recent research suggests the gut microbiome plays a role in T1DM, with dysbiosis contributing to its onset. Probiotics may help improve glycemic control and reduce inflammation, but their effects in children with T1DM are unclear. This study systematically reviews the impact of probiotics and related supplements on glycemic control in pediatric T1DM patients.

**Methods:**

This study adhered to PRISMA guidelines and was registered in PROSPERO (CRD42025633971). We searched databases including PubMed and EMBASE until January 5, 2025. The focus was on randomized controlled trials (RCTs) involving participants under 18 with T1DM, examining the effects of probiotics, prebiotics, and synbiotics on glycemic control indexes like fasting blood glucose (FBG), hemoglobin A1c (HbA1c), C-peptide, and insulin needs. Two researcher extracted data, quality was assessed via the Cochrane Handbook, and STATA 16 was used for statistical analysis.

**Results:**

Eight RCTs with 494 participants (246 intervention, 248 control) showed that probiotics and synbiotics significantly reduced HbA1c levels [Weighted Mean Difference (WMD) = −0.25%, 95% Confidence Interval (CI) = −0.45, −0.04; *p* = 0.019] with low heterogeneity [I-squared (I^2^) = 22%]. However, no significant changes were found in FBG, C-peptide levels, or insulin requirements. Sensitivity analyses yielded similar directions of effect for HbA1c. Subgroups suggested larger HbA1c reductions with longer intervention duration, shorter disease duration, and multi-strain formulations.

**Conclusion:**

Probiotic supplementation may achieve a small improvement in HbA1c in pediatric T1DM. Adequate dosing, longer intervention duration, and multi-strain formulations may be more likely to improve HbA1c, but the clinical importance is uncertain. However, our result shows no significant effects on fasting blood glucose, C-peptide, or insulin requirements; no routine clinical recommendations are proposed. The role of probiotics and related supplements in long-term glycemic control still requires confirmation through trials with extended follow-up. Large-scale, rigorously designed studies are needed to determine optimal intervention parameters, clarify underlying mechanisms, and evaluate the clinical applicability of probiotics in T1DM management.

**Systematic Review Registration:**

identifier [CRD42025633971].

## 1 Introduction

Type 1 diabetes mellitus (T1DM) is a chronic autoimmune disease characterized by immune-mediated destruction of pancreatic β-cells, eventually leading to absolute insulin deficiency (1). In recent years, the incidence of T1DM has been increasing globally, particularly among children and adolescents (2). According to the International Diabetes Federation (IDF) 2025 report, approximately 9.2 million people are living with T1DM worldwide, with more than 80% of cases occurring in individuals under the age of 20 (3). The development of T1DM typically progresses through multiple stages, beginning with genetic susceptibility and environmental triggers, followed by immune activation, islet inflammation, and progressive loss of β-cell function. Based on its natural history, T1DM can be divided into three clinical stages: Stage 1 involves the presence of two or more islet autoantibodies with normal blood glucose; Stage 2 includes abnormal glucose metabolism without overt symptoms; and Stage 3 corresponds to the clinical onset of diabetes, meeting diagnostic criteria. Some newly diagnosed patients experience a transient “honeymoon phase”, during which measurable C-peptide levels indicate residual β-cell function. Individuals with longer disease duration typically exhibit near-complete β-cell exhaustion and rely entirely on exogenous insulin (4). These disease-stage–related pathological differences may influence how patients respond to adjunctive therapeutic interventions. Despite advances in current treatment strategies, many individuals still struggle to achieve sustained glycemic control, highlighting the need for novel approaches to optimize long-term management of T1DM.

Recent research has underscored the potential role of the gut microbiome in the pathogenesis and progression of T1DM, as well as in various immune-mediated disorders (5). Accumulating evidence suggests that individuals with T1DM commonly exhibit significant gut microbial dysbiosis, characterized by reduced diversity, a decreased abundance of beneficial microbes, and an increased presence of pro-inflammatory bacteria (6, 7). Such an imbalance can provoke autoimmune responses against pancreatic β-cells through multiple mechanisms, thereby fostering chronic inflammation and impairing insulin secretion (8). One major pathway involves increased intestinal permeability, which allows bacterial endotoxins such as lipopolysaccharides (LPS) to translocate into systemic circulation. This translocation may activate innate immune responses and promote inflammation-driven β-cell destruction (9). Additionally, the depletion of short-chain fatty acids (SCFAs)—key microbial metabolites with anti-inflammatory and regulatory properties—may impair mucosal immune homeostasis and reduce the number or function of regulatory T cells (Tregs), further exacerbating immune dysfunction (10). Moreover, Early-life factors such as cesarean delivery, lack of breastfeeding, and antibiotic exposure may further exacerbate dysbiosis, potentially increasing the risk of T1DM onset (11, 12). Together, these findings highlight that the gut microbiome may not simply reflect immune dysfunction in T1DM, but actively contribute to its onset and progression. Consequently, strategies aimed at restoring gut microbial balance have been proposed as a promising adjunctive approach to modulate immune responses, improve gut barrier function, and reduce systemic inflammation in individuals with T1DM.

Probiotics, defined as live microorganisms that confer health benefits to the host, have garnered increasing interest for their potential to modulate the gut–immune–metabolic axis in type 1 diabetes mellitus (T1DM). A growing body of evidence suggests that probiotics may protect pancreatic β-cells and improve glycemic control through a series of interrelated mechanisms. By reshaping the gut microbial landscape—enhancing the abundance of beneficial taxa such as *Lactobacillus* and *Bifidobacterium* while suppressing proinflammatory species like *Escherichia coli*—probiotics help reduce systemic endotoxin exposure and attenuate low-grade inflammation associated with islet autoimmunity. This microbial modulation supports a more balanced immune environment, mitigating the chronic immune responses that underlie β-cell destruction (13). Additionally, probiotic administration has been linked to increased production of SCFAs, particularly butyrate, which reinforces intestinal epithelial barrier function and plays a vital role in immune tolerance. Butyrate and other SCFAs also exert direct effects on β-cells by improving mitochondrial activity, reducing oxidative stress, and upregulating insulin gene expression, thereby supporting residual β-cell function in the early or partial stages of disease (14). Beyond local effects in the gut, probiotics modulate systemic immunity by promoting regulatory T cell (Treg) expansion and fostering tolerogenic dendritic cell phenotypes, which collectively downregulate pathogenic Th1/Th17 responses implicated in T1DM progression (15). These concerted actions not only reduce inflammatory burden but may also delay β-cell failure and extend the therapeutic window for intervention. Synbiotics and prebiotics, which further promote a favorable gut environment, are being explored as adjunctive strategies to sustain these benefits over time.

While some studies indicate potential benefits of probiotics for patients with T1DM, the majority of research has predominantly centered on adult populations, yielding inconsistent findings (16–18). Furthermore, there exists considerable debate regarding the effects of probiotics on glycemic control in pediatric and adolescent populations (19, 20). Research regarding the effects of probiotics on blood glucose regulation in pediatric T1DM remains in its preliminary stages, necessitating further systematic investigations to substantiate the clinical efficacy of probiotics in diabetes management. Consequently, this study conducted a systematic review and meta-analysis to assess the effects of probiotics and related supplements on glycemic control in children and adolescents with T1DM. In addition to assessing key clinical outcomes such as HbA1c, fasting blood glucose, C-peptide levels, and insulin requirements, we also performed detailed subgroup analyses to account for potential sources of heterogeneity across studies. This comprehensive approach aim to provide more definitive evidence to inform clinical treatment strategies.

## 2 Materials and methods

### 2.1 Protocol validation

The Preferred Reporting Items for Systematic Reviews and Meta-Analyses (PRISMA) Statement served as a guideline for the conduct and reporting of this systematic review and meta-analysis (21). Additionally, this systematic review has been registered in the International Prospective Register of Systematic Reviews (PROSPERO) database (Registration number: CRD42025633971).

### 2.2 Literature search

The following electronic databases were independently searched by two researchers until January 5, 2025: PubMed, EMBASE, Web of Science, ScienceDirect, and the English Clinical Trial Registry. Additionally, published reviews and their references were manually examined to identify any further studies that met the inclusion criteria. A combination of Medical Subject Headings (MeSH) terms and free-text keywords was employed, including terms such as “type 1 diabetes”, “probiotics”, “prebiotics”, “synbiotics”, and “randomized controlled trials”. Boolean operators were utilized to enhance sensitivity (“OR”) and precision (“AND”), tailored to the specific syntax of each database. For instance, the search strategy implemented in PubMed was formulated as follows: [“Diabetes Mellitus, Type 1” (Mesh) OR Type 1 Diabetes (Title/Abstract)] AND [“Probiotics” (Mesh) OR Probiotic (Title/Abstract) OR “Probiotics” (Mesh) OR “Prebiotics” (Mesh) OR Prebiotic (Title/Abstract) OR “Synbiotics” (Mesh) OR Synbiotic (Title/Abstract)]. Supplementary Table S1 of the Supplementary Materials presents the PubMed search strategies as a representative example.

### 2.3 Selection criteria

A study was included if the following criteria were met: (1) RCT; (2) T1DM patients (children <18 years old); (3) interventions were limited to probiotics, prebiotics, and synbiotics with no requirement on duration; (4) written in the English language; and (5) providing parameters of glycemic control, such fasting blood glucose (FBG), hemoglobin A1c (HbA1c), C-peptide, and insulin requirements.

The exclusion criteria were as follows: (1) the subjects had other types of disease; (2) the probiotics were taken within three months before the trial; (3) Crucial data are incomplete; and (4) review papers, case-control studies, medical hypotheses, letters to the Editor, and duplicate studies.

### 2.4 Data extraction

Data were extracted by one author (H.H.) and independently verified by a second author (DY.M.). Any discrepancies were resolved by discussion or with a third author (LP.W.). The following information was extracted from each eligible study: (1) the surname of the first author, (2) the year of publication, (3) participant characteristics, including number, gender, and age, (4) study design, (5) duration and dosage of probiotics or related supplements administered, and (6) primary measured outcomes. In instances where data were unclear or incomplete, the data analyst reached out to the corresponding authors via email to request additional information. If a response was not received, a second attempt to contact the authors was made. Should there be continued non-response following the second attempt, the study was excluded from the analysis.

### 2.5 Quality assessment

The Cochrane Handbook was utilized by two reviewers to evaluate the risk of bias in each study. This assessment encompassed seven domains: random sequence generation (selection bias), allocation concealment (selection bias), blinding of participants and personnel (performance bias), blinding of outcome assessment (detection bias), incomplete outcome data (attrition bias), selective reporting (reporting bias), and other biases. The levels of bias were classified as “Low risk”, “High risk”, or “Unclear risk”. Any discrepancies that arose were addressed through discussions involving a third assessor.

The certainty of the evidence for the primary outcomes was evaluated using the GRADE (Grading of Recommendations Assessment, Development, and Evaluation) approach, based on five domains: risk of bias, inconsistency, indirectness, imprecision, and publication bias. The quality of evidence was classified into four categories, namely high, moderate, low, and very low, according to the corresponding evaluation criteria.

### 2.6 Statistical analysis

The meta-analysis was conducted using STATA software version 16 (Stata Corp LP). Extracted data were input into the software as mean differences accompanied by standard deviations (m ± SD). The mean difference (MD), standard deviation (SD), and 95% confidence interval (CI) served as the primary effect size indicators. In instances where data were initially reported as medians with interquartile ranges (IQR) or as lower and upper quartiles (Q1; Q3), skewness was evaluated using the online resource (https://www.math.hkbu.edu.hk/∼tongt/papers/median2mean.html) (22, 23). If the data exhibited no significant skewness, transformation to mean with SD was performed. Between-study heterogeneity was assessed using the I-squared [I^2^] statistic, with I^2^ values of 25%, 50%, and 75% indicating low, moderate, and high heterogeneity, respectively (24). In cases of significant heterogeneity (I^2^ > 50%, *P* < 0.05), a random-effects model was utilized. Additionally, Sensitivity analyses were undertaken to evaluate the impact of the following strategies on the pooled effects: leave-one-out analysis, exclusion of studies at high risk of bias, and exclusion of trials involving prebiotic-only interventions. Subgroup analyses were subsequently performed by intervention duration, disease duration, and probiotics formulation.

## 3 Results

### 3.1 Search details

**Figure 1:**
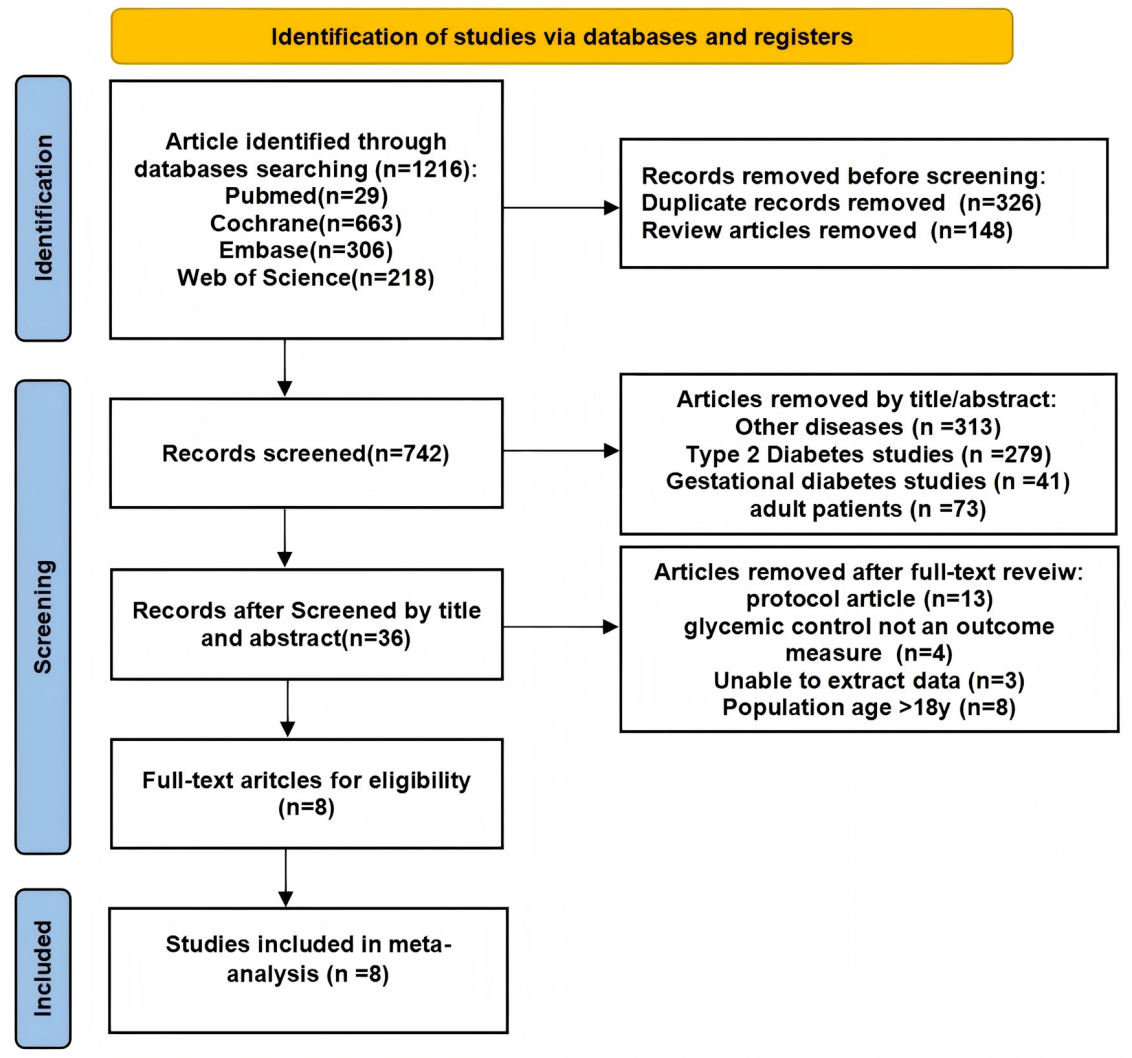
Flow chart of study selection.

The literature review resulted in the identification of 1,216 records, of which 474 were excluded: 326 due to duplication and 148 classified as review articles. Following an evaluation of the titles and abstracts, an additional 706 papers were eliminated on the basis of their focus on unrelated diseases (e.g., type 2 diabetes mellitus). Ultimately, the number of articles was narrowed down to 36, which underwent a thorough review, leading to the acquisition and reassessment of their full-text versions. From this final selection, 8 articles were included in the meta-analysis (25–32) (Figure 1).

### 3.2 Study characteristics

Table 1 outlines the general characteristics of the eight included randomized controlled trials (RCTs). To facilitate a clearer understanding of patient heterogeneity, Supplementary Table S2 presents detailed baseline and post-intervention clinical data for each study (25–32). A total of 494 participants were enrolled, comprising 246 individuals in the intervention group and 248 in the control group. The mean age of participants was 10.99 years, with the duration of Type 1 Diabetes (T1D) ranging from less than 2 months to 7.31 years. All clinical trials included both male and female participants, maintaining a balanced male-to-female ratio of 1.3. In terms of interventions, one study utilized a prebiotic (inulin) (25), another employed a single-strain probiotic (Lactobacillus rhamnosus GG) (26), while six studies implemented multistrain probiotic or synbiotic supplements (27–32). Significantly, one study investigated the Collaborative effects of probiotics and the influenza vaccine on immune function and HbA1c levels in patients with T1DM (26). To reduce confounding effects and measurement bias, data were extracted prior to the administration of the influenza vaccine, specifically within the first three months of probiotic treatment. Four studies administered probiotics and related supplements for a duration of three months (25, 26, 28, 31), with one study reporting a duration of less than three months (27) and three studies extending beyond three months (29, 30, 32). Probiotics and related supplements were delivered in various forms, including capsules (25, 28–31), drops (26), and powder (27, 32).

**Table 1:** Characteristics of included studies.

Author/Year	Country	Sample Size	Age	Male/Female	Duration of T1DM(Year)	BMI (kg/m^2^)	Insulin dose (U/kg/d)	Intervention; Control	Dose	Intervention Duration	Main measured markers
Ho et al. (25)	Canada	Prebiotic: 17	12.5 ± 2.76	12/5	7.31 ± 3.93	NA	0.87 ± 0.25	Prebiotic capsule: oligofructose enriched inulin (chicory root-derived)	8 g/capsule, QD	3 months	HbA1c, C-peptide
		Control: 21	11.94 ± 2.61	7/14	4.70 ± 3.07	NA	0.92 ± 0.29	Placebo capsule: maltodextrin	8 g/capsule, QD		
Bianchini et al. (26)	Italy	Probiotic: 34	13.45 ± 4.67	25/9	NA	NA	NA	Probiotic drop: *Lactobacillus rhamnosus* GG	5 × 109 LGG/drops, BID	3 months	HbA1c
		Control: 30	13.09 ± 4.66	19/11	NA	NA	NA	Placebo drop: with a similar formulation but not containing probiotic	5 drops, BID		
Zare et al. (27)	Iran	Synbiotic: 22	10.36 ± 2.53	11/11	4.45 ± 1.96	19.77 ± 4.18	NA	Synbiotic powder: *Lactobacillus sporogenes* GBI-30, maltodextrin, and fructooligosaccharide	2 g powder (109 CFU), QD	2 months	HbA1c, FBG
		Control: 22	10.04 ± 2.08	10/12	4.04 ± 1.36	17.86 ± 3.45	NA	Placebo powder: starch	2 g powder, QD		
Kumar et al. (28)	India	Probiotic: 47	7.92 ± 3.92	28/19	<2 months	15.2 ± 1.8	1.0 ± 0.54	Multistrain probiotic capsule: *L.paracasei* DSM 24733, *L.plantarum* DSM 24730, *L.acidophilus* DSM 24735, and *L.delbrueckii* subsp.bulgaricus DSM 24734, *B.longum* DSM 24736, *B.infantis* DSM 24737, *B.breve* DSM 24732, and *Streptococcus thermophilus* DSM 24731	1.125 × 1011 bacteria/capsule, QD	3 months	HbA1c, C-peptide, Insulin Requirement
		Control: 49	9.1 ± 4.95	28/21	<2 months	15.7 ± 2.3	0.9 ± 0.46	Placebo capsule: microcrystalline cellulose	1 capsule, QD		
Groele et al. (29)	Poland	Probiotic: 48	12.31 ± 2.13	25/23	<2 months	0.20 ± 0.84(SDS)	0.29 ± 0.17	Multistrain probiotic capsule: *L.rhamnosus* GG ATCC 53103 and *B.lactis* Bb12 DSM 15954	109 CFU/capsule, QD	6 months	HbHbA1c, C-peptide, Insulin Requirement
		Control: 48	13.17 ± 2.59	30/18	<2 months	0.24 ± 1.02(SDS)	0.36 ± 0.2	Placebo capsule: maltodextrin	1 capsule, QD		
Wang et al. (30)	China	Probiotic: 27	14.1 ± 5.1	13/14	6.2 ± 4.47	NA	0.8 ± 0.3	Multistrain probiotic capsule: 1:1 mixture ratio of *Lactobacillus salivarius* subsp.salicinius AP-32, *L.johnsonii* MH-68, and *Bifidobacterium animalis* subsp.lactis CP-9	5 × 109 CFU/capsule, QD	6 months	HbA1c
		Control: 29	14.3 ± 4.6	19/10	6.49 ± 3.91	NA	0.8 ± 0.3	Placebo: insulin therapy			
Shabani-Mirzaee (31)	Iran	Probiotic: 26	9.6 ± 3.5	14/12	6 months—3 years	16.1 ± 1.9	<7 years old 0.3–0.6; ≥ 7 years old 0.7–1	Multistrain probiotic capsule: *Bifidobacteriumlactis* over 5.3 billion, *Bifidobacteriumbifidum* over 1 billion, *Lactobacillus acidophilus* over 1 billion, and *Lactobacillus ramenus* over 5 billion	1 capsule, QD	3 months	HbA1c, FBG
		Control: 26	9.4 ± 3.0	13/13	6 months—3 years	16.0 ± 2.4	<7 years old 0.3–0.6; ≥ 7 years old 0.7–1	Placebo: insulin therapy			
Lokesh et al. (32)	India	Probiotic: 30	7.44 ± 3.42	17/13	<2 months	14.82 (3.4)	1.07 ± 0.49	Synbiotic powder: *Streptococcus thermophilus* DSM 24731, *Bifidobacteria infantis* DSM 24737, *Bifidobacteria longum* DSM 24736, *Bifidobacteria breve* DSM 24732, *Lactobacillus acidophilus* DSM 24735, *Lactobacillus plantarum* DSM 24730, *Lactobacillus paracasei* DSM 24733, *Lactobacillus. delbrueckii* subsp.bulgaricus DSM 24734 in a base of corn starch	1 sachet, QD	6 months	HbA1c, FBG, C-peptide, Insulin Requirement
		Control: 30	7.08 ± 3.77	15/15	<2 months	14.82 (3.4)	1.14 ± 0.47	Placebo powder: corn starch	1 sachet, QD		

NOTES: Normally distributed quantitative variables are presented as mean ± SD.

NA, not available; SDS, standard deviation scores; BID, twice daily; QD, once daily; CFU, colony forming unit; HbA1c, hemoglobin A1c; C-peptide, fasting C-peptide; FBG, fasting blood glucose; DIU, daily insulin usage; BMI, body mass index.

### 3.3 Quality assessment

**Figure 2:**
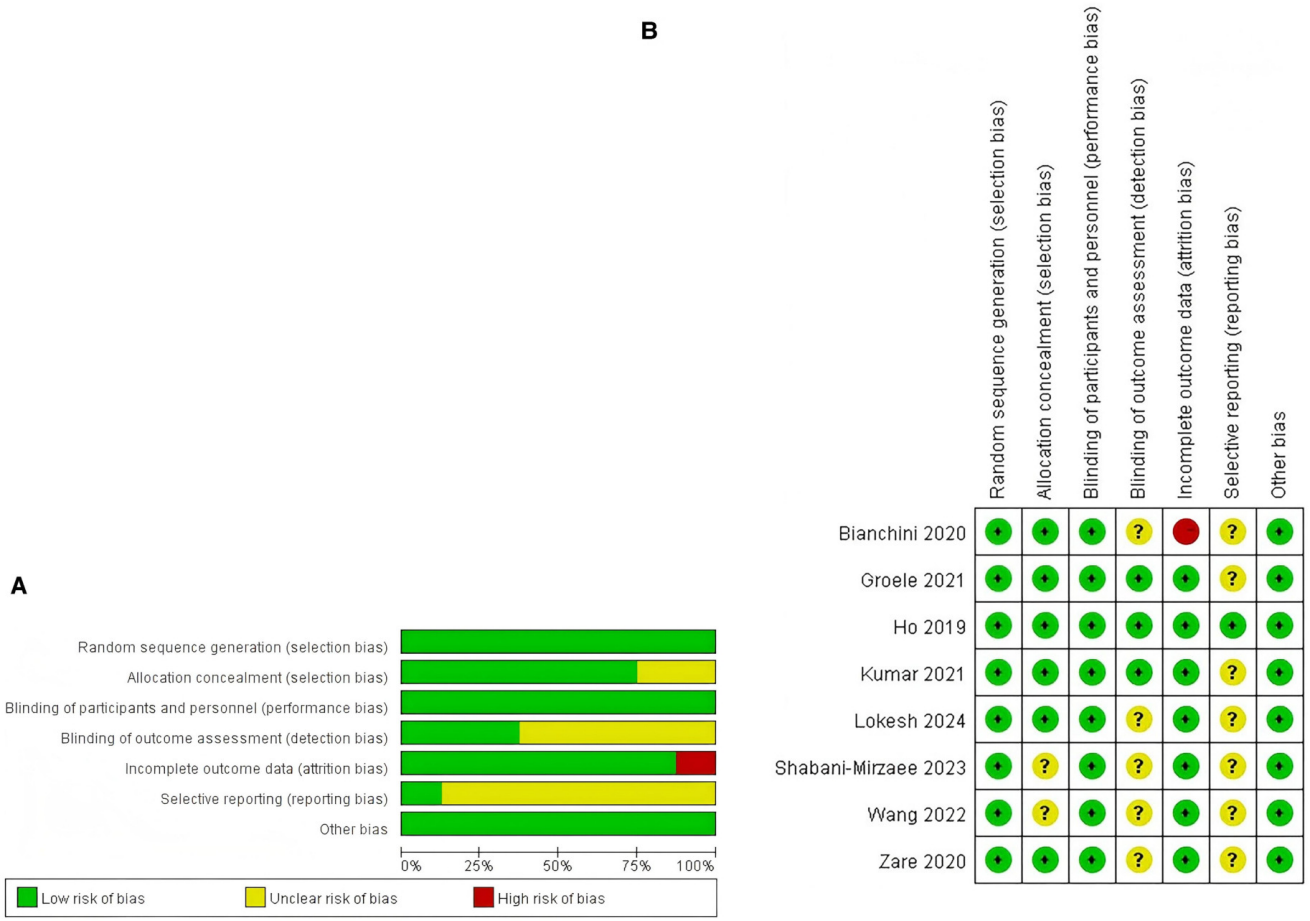
Risk-of-bias summary. **(A)** Risk of bias summary. **(B)** Risk of bias graph.

Within the studies incorporated into the analysis, one study was classified as exhibiting a high risk of bias (12.5%, 1/8), primarily attributable to incomplete outcome data (26). Furthermore, six studies (75%, 6/8) were determined to have an unclear risk of bias (27–32), while one study (12.5%, 1/8) was evaluated as having a low risk of bias (25). Additional information regarding the quality assessment is presented in Figures 2A,B, with further details provided in Supplementary Table S3.

An evaluation of evidence quality using the GRADE approach is presented in Supplementary Figure S1. The certainty of evidence for HbA1c was rated low, with downgrades for risk of bias (serious) and imprecision (serious) (8 trials; total *n* = 497) (25–32). For FBG, C-peptide, and insulin requirements, the certainty was rated very low due to risk of bias (serious) and inconsistency (serious) together with imprecision (very serious) [FBG: 3 trials, total *n* = 156 (27, 31, 32); C-peptide: 4 trials, total *n* = 278 (25, 28, 29, 32); insulin: 3 trials, total *n* = 240 (28, 29, 32)]. No additional downgrading was applied for other considerations (e.g., publication bias); however, given the small number of trials, small-study/publication bias cannot be excluded.

### 3.4 Results of meta-analysis

#### 3.4.1 Baseline consistency analysis

Before performing the meta-analysis, baseline consistency between the two groups was confirmed to ensure the validity of subsequent analyses.

The results of the baseline assessment, detailed in Table 2 and Supplementary Figure S2, reveal no statistically significant differences between the groups concerning HbA1c [Weighted Mean Difference (WMD) = −0.02%; 95% CI = −0.28, 0.24; *p* = 0.871], FBG (WMD = 16.41 mg/dl; 95% CI = −1.05, 33.86; *p* = 0.065), C-peptide levels (WMD = 0.04 ng/ml; 95% CI = −0.07, 0.16; *p* = 0.447), and insulin requirements (WMD = −0.05 Units/kg/day; 95% CI = −0.12, 0.02; *p* = 0.146). These results indicate a lack of baseline differences, thereby justifying the continuation of the meta-analysis.

**Table 2:** Baseline consistency analysis.

Meta-analyzed variables	Heterogeneity		Effect model	Meta analysis results		
	I^2^	*P*		WMD	95% CI (%)	*P*
HbA1c	47.5%	0.064	Fixed-effects model	−0.02	(−0.28, 0.24)	0.871
FBG	0.0%	0.484	Fixed-effects model	16.41	(−1.05, 33.86)	0.065
C-peptide	0.0%	0.870	Fixed-effects model	0.04	(−0.07, 0.16)	0.447
Insulin requirements	13.0%	0.317	Fixed-effects model	−0.05	(−0.12, 0.02)	0.146

#### 3.4.2 Effect of probiotics and related supplements on HbA1c

**Figure 3:**
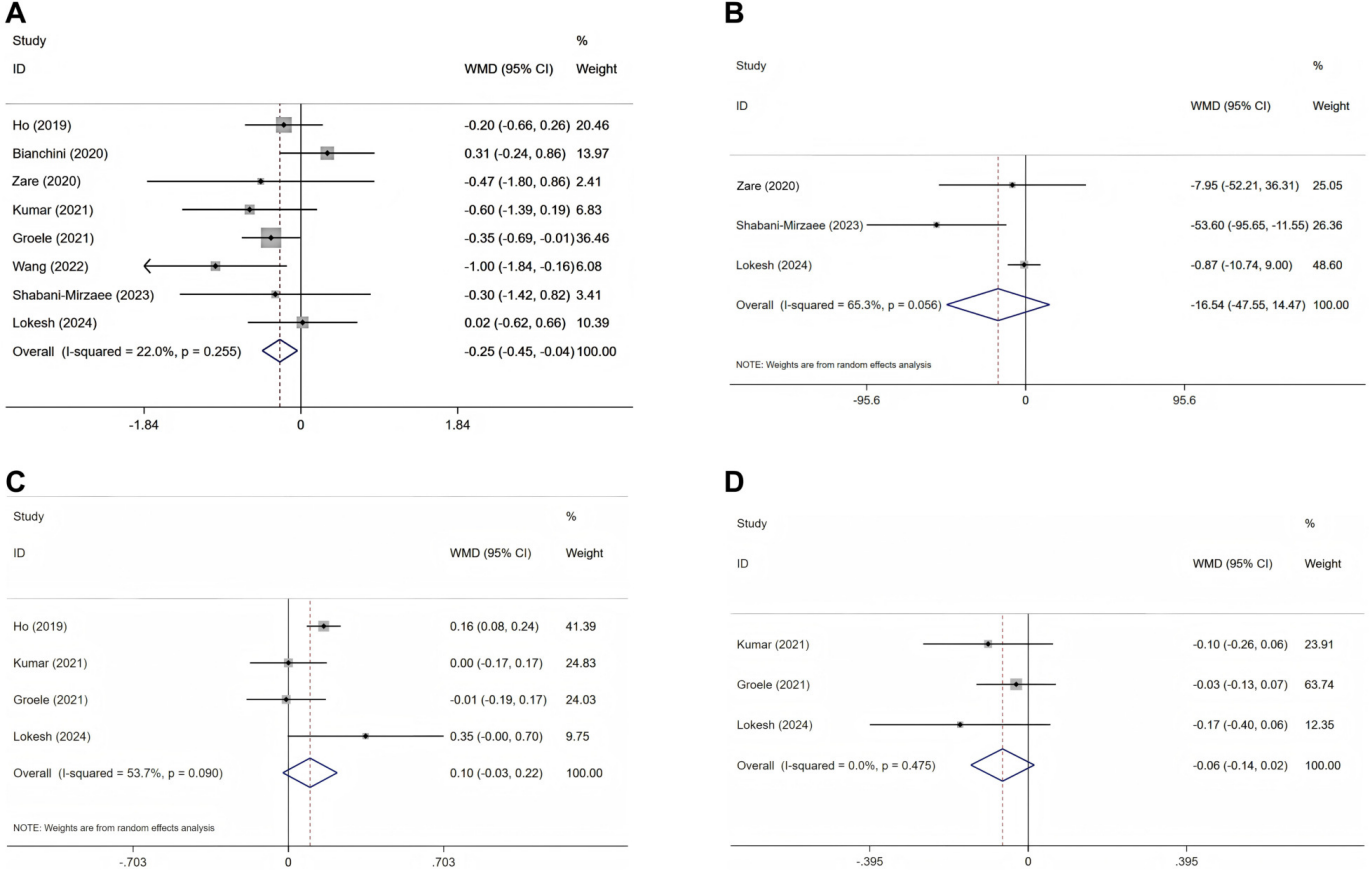
Forest plots of randomised controlled trials investigating the efficacy of probiotics on T1DM; selected variables are HbA1c **(A)**, FBG **(B)**, C-peptide **(C)**, and insulin requirements **(D)**.

The effectiveness of probiotics and related supplements on HbA1c levels was assessed in eight studies (25–33), encompassing a total of 494 participants (intervention group: 246; control group: 248). The pooled analysis revealed that probiotics and synbiotic supplementation resulted in a significant reduction in HbA1c levels (WMD = −0.25%; 95% CI = −0.45, −0.04; *p* = 0.019), exhibiting low heterogeneity (I^2^ = 22%; *p* = 0.255) (Figure 3A and Table 3).

**Table 3:** Results of meta-analyzed variables.

Meta-analyzed variables	Heterogeneity		Effect model	Meta analysis results		
	I^2^	*P*		WMD	95% CI (%)	*P*
HbA1c	22.0%	0.255	Fixed-effects model	−0.25	(−0.45, −0.04)	0.019
FBG	65.3%	0.056	Random-effects model	−16.54	(−47.55, 14.47)	0.296
C-peptide	53.7%	0.090	Random-effects model	0.10	(−0.03, 0.22)	0.119
Insulin requirements	0.0%	0.475	Fixed-effects model	−0.06	(−0.14, 0.02)	0.113

#### 3.4.3 Effect of probiotics and related supplements on FBG

In the pooled analysis of three studies (27, 31, 32) involving a total of 156 participants (with 78 in the intervention group and 78 in the control group), no statistically significant effect of probiotics and prebiotics on FBG was observed (WMD = −16.54 mg/dl; 95% CI = −47.55–14.47; *p* = 0.296). Furthermore, the results demonstrated considerable heterogeneity (I^2^ = 65.3%, *p* = 0.056) (Figure 3B and Table 3).

#### 3.4.4 Effect of probiotics and related supplements on C-peptide

The comprehensive analysis revealed no statistically significant enhancement in serum C-peptide concentrations attributable to probiotics and associated supplementation (WMD = 0.10 ng/ml; 95% CI = −0.03, 0.22; *p* = 0.119) following the evaluation of four RCTs involving a total of 278 participants (intervention group: 137; control group: 141) (25, 28, 29, 32). Additionally, notable heterogeneity among the studies was identified (I^2^ = 53.7%, *p* = 0.090) (Figure 3C and Table 3).

#### 3.4.5 Effect of probiotics and related supplements on insulin requirements

Three RCTs (28, 29, 32) investigated the impact of probiotic administration on daily insulin requirements, involving a total of 240 participants (120 in the intervention group and 120 in the control group). The analysis revealed no significant association between probiotic and synbiotic supplementation and insulin requirements, with a WMD of −0.06 Units/kg/day(95% CI = −0.14, 0.02; *p* = 0.113). Additionally, no heterogeneity was detected among the studies (I^2^ = 0.0%, *p* = 0.475) (Figure 3D and Table 3).

### 3.5 Sensitivity analysis

In conducting the sensitivity analysis, we utilized a leave-one-out exclusion method to evaluate the outcome indicators of HbA1c, FBG, C-peptide, and insulin requirements. Each study was systematically excluded in turn prior to the calculation of pooled effect sizes. The findings from the sensitivity analysis revealed no significant variations, suggesting that the pooled effect sizes in this meta-analysis are both stable and reliable, as demonstrated in Supplementary Figure S3. It is important to note that a subgroup analysis was not conducted due to the limited number of studies available.

For the outcome of HbA1c, two sensitivity analyses were performed. After removing one high risk-of-bias study (26), the pooled effect was WMD −0.34% (95% CI −0.56 to −0.11; I^2^ = 0%; Supplementary Figure S4). Compared with the main analysis (WMD −0.25%, 95% CI −0.45 to −0.04; I^2^ = 22%), the direction was consistent with a slightly larger effect and heterogeneity reduced to 0%, indicating that the results are not sensitive to this study and that the primary conclusion is more stable. After excluding the prebiotic-only trial (25), the pooled effect for HbA1c was WMD −0.26% (95% CI −0.49 to −0.03; I^2^ = 32.7%; Supplementary Figure S5). Relative to the main analysis, the direction remained consistent and the magnitude was similar, indicating that the overall HbA1c effect is not driven by the prebiotic study.

### 3.6 Subgroup analysis

**Figure 4:**
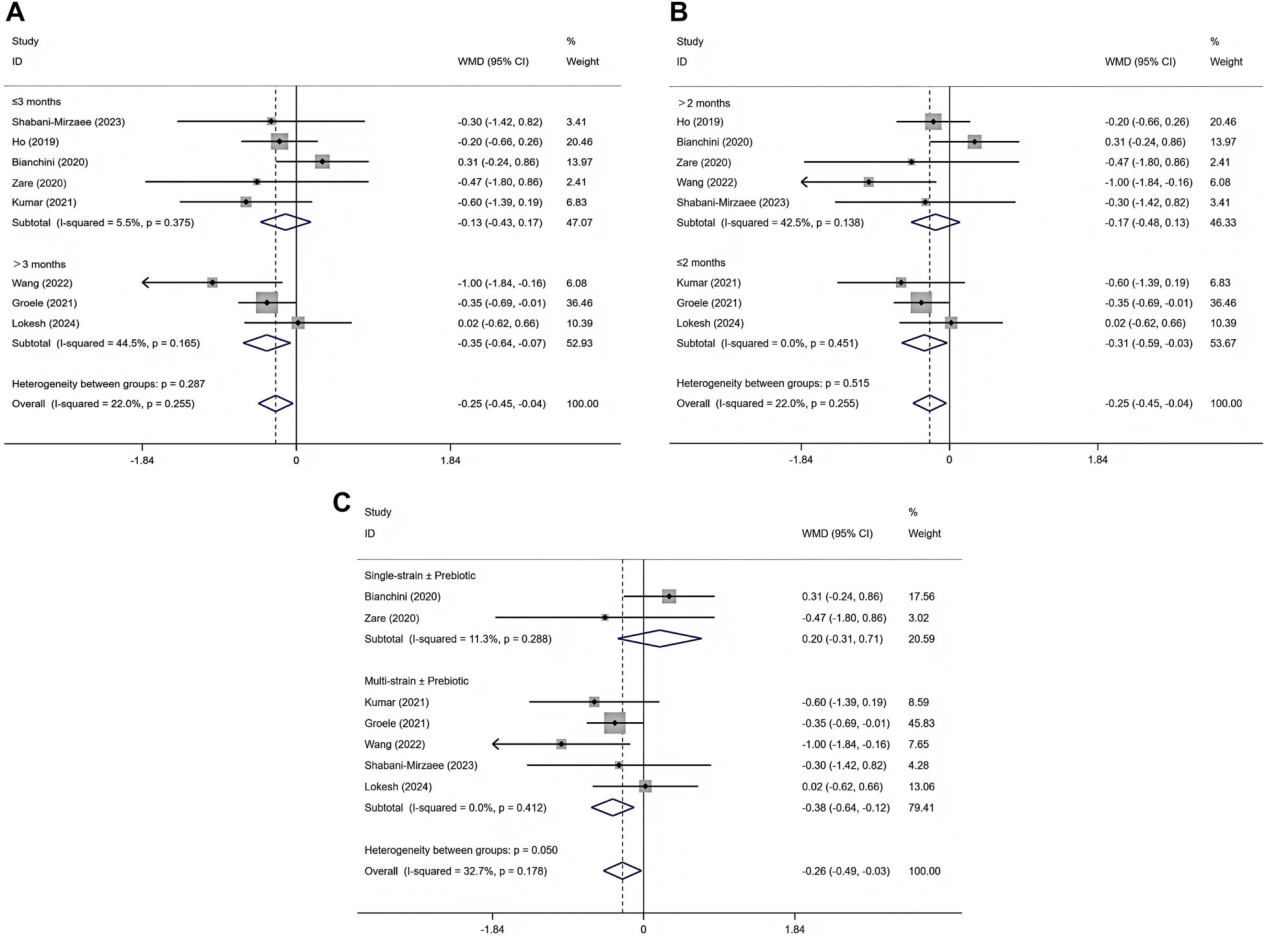
Subgroup analysis of intervention duration effects on HbA1c **(A)**, subgroup analysis of disease duration effects on HbA1c **(B)**, subgroup analysis of probiotics formulation on HbA1c **(C)**.

Subgroup analyses were not performed for FBG, C-peptide, or insulin requirement due to the limited number of eligible studies and small sample sizes. Subgroup analysis was conducted only for glycated hemoglobin HbA1c. When stratified by intervention duration, studies with a duration longer than 3 months showed a significant reduction in HbA1c [WMD = −0.35, 95% CI (−0.64, −0.07), *p* = 0.015] (29, 30, 32), whereas no significant effect was observed in studies with an intervention of 3 months or less [WMD = −0.13, 95% CI (−0.43, 0.17), *p* = 0.405] (25–28, 31) (Figure 4A). Heterogeneity was low in both subgroups (I^2^ = 44.5% for >3 months and 5.5% for ≤3 months), and the test for subgroup differences did not indicate a statistically significant interaction (*p* = 0.287). When stratified by disease duration, a significant reduction in HbA1c was observed among patients with a diagnosis of ≤2 months [WMD = −0.31, 95% CI (−0.59, −0.03), *p* = 0.031] (28, 29, 32), while no significant effect was found in those with a diagnosis of >2 months [WMD = −0.17, 95% CI (−0.48, 0.13), *p* = 0.264] (25–27, 30, 31) (Figure 4B). Heterogeneity was low in both subgroups (I^2^ = 0.0% for diagnosis ≤2 months and 42.5% for diagnosis >2 months), with no statistically significant difference between subgroups (*p* = 0.515) (Supplementary Tables S4, S5). Stratifying by probiotic formulation, the multistrain ± prebiotic subgroup (5 trials; 79.41% weight) showed WMD −0.38% (95% CI −0.64 to −0.12; I^2^ = 0%) (28–32), indicating lower HbA1c with minimal heterogeneity. The single-strain ± prebiotic subgroup (2 trials; 20.59% weight) showed WMD +0.20% (95% CI −0.31 to +0.71; I^2^ = 11.3%) (26, 27).The overall pooled effect across studies was WMD −0.26% (95% CI −0.49 to −0.03; I^2^ = 32.7%) (26–32). The between-subgroup heterogeneity test was *p* = 0.050 (Figure 4C and Supplementary Table S6).

## 4 Discussion

This systematic review and meta-analysis focused on the adjunctive role of probiotics and related supplements in the management of T1DM among children and adolescents. With growing evidence on the relationship between gut microbiota and metabolic diseases, modulation of the gut microbiome has been recognized as a promising strategy in diabetes care. Following a systematic search and rigorous screening of the literature, eight randomized controlled trials involving pediatric and adolescent T1DM populations were included. Given the small number of trials, we did not perform Egger or Begg tests (underpowered and potentially misleading in small-k settings)Risk of bias (ROB) assessed using the Cochrane Handbook/ROB 2 framework indicated only one low-risk study, six with some concerns, and one at high risk; overall study quality was limited. According to GRADE, the certainty of evidence was low for HbA1c and very low for fasting blood glucose (FBG), C-peptide, and insulin requirements, warranting cautious interpretation. Pooled analyses showed a modest mean reduction in HbA1c (approximately 0.25%), with no consistent improvements in FBG, C-peptide, or insulin requirements. Given that a commonly cited threshold for clinically meaningful change in HbA1c is about 0.5% (34), the observed magnitude suggests limited clinical importance. Sensitivity analyses (excluding the high–risk-of-bias trial, leave-one-out analyses, and excluding prebiotic-only trials) yielded broadly consistent directions of effect on HbA1c but did not materially raise the certainty of evidence. These findings differ from some meta-analyses conducted in type 2 diabetes mellitus (T2DM) or gestational diabetes mellitus (GDM) (35–39), suggesting that T1DM may have distinct pathophysiological characteristics and responses to microbiota-targeted interventions. Importantly, our pooled estimate integrates heterogeneous probiotic regimens (strain composition and co-administration of prebiotics) and does not attribute effects to any specific strain or formulation; the implicit exchangeability assumption across formulations may not hold biologically. In view of the limited evidence base and between-study variability, overall certainty remains low, and routine clinical recommendations are not proposed.

Prior evidence suggests that when the intervention duration approaches or spans the HbA1c assessment window (approximately 2–3 months), reductions are more likely to be observed, whereas shorter follow-up may fail to capture changes (40). Accordingly, we conducted an exploratory subgroup analysis by duration: longer courses tended to show signals of HbA1c reduction, whereas shorter courses did not. To further parse heterogeneity, two additional exploratory subgroupings were performed. First, by disease duration (newly diagnosed/shorter duration vs. longer duration), the former more often showed favorable changes in HbA1c; however, definitions and reporting of “disease duration” varied across studies, limiting interpretability. Second, by probiotic formulation (single-strain vs. multi-strain), subgroup directions were broadly consistent, with multi-strain formulations showing a tendency toward larger HbA1c reductions; nonetheless, confidence intervals were wide and the exchangeability assumption across formulations was unverified, precluding comparative effectiveness inferences. All three subgroup analyses were exploratory with limited statistical power and do not support firm stratified conclusions.

At the mechanistic level, probiotics may influence glycemic control through multiple pathways. Microbiota-directed effects include increased abundance of beneficial taxa (e.g., Lactobacillus, Bifidobacterium), reductions in proinflammatory taxa (e.g., Proteobacteria), and restoration of diversity and homeostasis (41). Metabolically, increased short-chain fatty acid production (notably butyrate and propionate) may enhance gut barrier integrity via tight junction upregulation, attenuate translocation of lipopolysaccharide, and reduce low-grade inflammation (42–44). Immunomodulatory effects may include promotion of regulatory T-cell differentiation, suppression of Th1/Th17 activity, and a shift toward anti-inflammatory cytokine profiles, thereby potentially limiting immune-mediated β-cell injury. From a metabolic signaling perspective, short-chain fatty acids can activate G-protein–coupled receptors, stimulate GLP-1 secretion, slow gastric emptying, and improve insulin secretion and sensitivity (45), with potential antioxidant effects via enhanced antioxidant enzyme activity (46). These mechanisms, synthesized from the broader literature, represent plausible pathways that could partially account for the signal observed with multi-strain regimens; however, the included RCTs did not directly or systematically measure mechanistic endpoints, and these explanations remain hypothesis-generating.

Probiotic regimens showed no significant effects on FBG, C-peptide, or insulin requirements in children and adolescents with T1DM. This may reflect disease-specific pathophysiology: unlike T2DM, most individuals with T1DM have profound β-cell destruction and depend on exogenous insulin, so improvements in insulin sensitivity may be insufficient to produce measurable changes in glycemia among insulin-dependent patients (47). Moreover, probiotic effects may preferentially influence longer-term glycemic averages (HbA1c) rather than single time-point FBG or direct β-cell function indices such as C-peptide (48, 49). Statistically, the higher heterogeneity observed for FBG and C-peptide may stem from differences in formulations (e.g., prebiotics vs. multi-strain synbiotics), dosing, disease duration, and age distributions. Although most studies reported no statistically significant baseline imbalances, some baseline estimates (e.g., FBG) exhibited wide confidence intervals (e.g., −1.05–33.86 mg/dl), suggesting potential residual imbalance or measurement error that could affect the stability of effect estimates. Given the limited number of studies per outcome, we did not pursue additional subgroup analyses for these endpoints and downgraded GRADE for inconsistency and imprecision. No clinical recommendations are proposed for these outcomes on current evidence. This study has several limitations: (1) small overall sample size with uneven study quality and limited power; (2) marked clinical and methodological heterogeneity (strain composition and co-administration of prebiotics, dosing and duration, disease stage, outcome measurement and aggregation), with pooled estimates contingent on an exchangeability assumption across distinct formulations, limiting comparative effectiveness and causal inference; (3) generally short follow-up, with insufficient evidence on long-term efficacy and safety; (4) incomplete reporting on domains pertinent to risk of bias, including one high-risk study; (5) too few studies to perform robust publication bias assessments, so small-study and reporting biases cannot be excluded; and (6) wide confidence intervals in baseline estimates for some outcomes, suggesting potential residual imbalance. Overall, GRADE certainty ranged from low to very low. We therefore do not advance routine clinical recommendations. Therefore, future research should address these issues from several perspectives. First, larger sample sizes and multicenter collaborative studies employing rigorously designed RCTs with detailed subgroup analyses are warranted to elucidate the effects and underlying mechanisms of probiotic interventions on both long-term and short-term glycemic control in diverse patient populations. Second, refined selection of probiotic strains and dosage optimization represent critical directions for future investigations. Systematic comparisons of different strains, combinations, dosages, and intervention durations are necessary to provide a scientific basis for personalized treatment regimens. Third, exploring the synergistic effects of probiotics in conjunction with conventional hypoglycemic agents, dietary modifications, and exercise interventions may facilitate the development of a more comprehensive T1DM management strategy. Additionally, further basic research is required to elucidate the mechanisms by which probiotics influence glucose metabolism through modulation of gut microbiota composition, enhancement of intestinal barrier function, reduction of systemic inflammation, and regulation of short-chain fatty acid metabolism, as well as to develop predictive biomarkers. Finally, comprehensive evaluations of the long-term safety and tolerability of probiotic supplementation are essential to provide the necessary data for their clinical application.

## 5 Conclusion

This systematic review and meta-analysis observed a small, statistically significant reduction in HbA1c (≈0.25%) with probiotic supplementation in children and adolescents with type 1 diabetes; however, the certainty of this evidence is low, the clinical importance is uncertain (below commonly cited ≈0.5% thresholds), and no consistent effects were detected for fasting blood glucose, C-peptide, or insulin requirements. The evidence base is constrained by small sample sizes, risk of bias, and substantial clinical and methodological heterogeneity across strains, dosages, regimens, and follow-up, and too few trials to reliably assess publication bias. Sensitivity analyses showed broadly similar directions of effect but did not increase the GRADE certainty. While adequate dosing and longer intervention duration may favor gut colonization and downstream metabolic effects, whether probiotics can sustain long-term glycemic control remains unproven and requires trials with extended follow-up. Given the unverified exchangeability of different formulations, routine clinical recommendations are not proposed. Future research should include larger, rigorously designed multicenter RCTs using standardized, well-characterized strains with prespecified dosing and duration, ideally with head-to-head comparisons and mechanistic biomarker collection, to clarify comparative efficacy, underlying mechanisms, and optimized treatment protocols for T1DM management.

## Data Availability

The datasets analyzed for this study are available from the corresponding author upon reasonable request.

## References

[1] Wang JF Lee MS Tsai TL Leiferman EM Trask DJ Squire MW Bone morphogenetic protein-6 attenuates type 1 diabetes mellitus-associated bone loss. Stem Cell Transl MED. (2019) 8:522–34. 10.1002/sctm.18-0150 PMC6525561 30784225

[2] Stanescu DE Lord K Lipman TH. The epidemiology of type 1 diabetes in children. Endocrin Metab Clin. (2012) 41:679–94. 10.1016/j.ecl.2012.08.001 23099264

[3] Owens DR Gurudas S Sivaprasad S Zaidi F Tapp R Kazantzis D IDF diabetes atlas: a worldwide review of studies utilizing retinal photography to screen for diabetic retinopathy from 2017–2024 inclusive. Diabetes Res Clin PR. (2025) 226:112346. 10.1016/j.diabres.2025.112346 40578519

[4] Mittal M Porchezhian P Kapoor N. Honeymoon phase in type 1 diabetes mellitus: a window of opportunity for diabetes reversal? World J Clin Cases. (2024) 12:9–14. 10.12998/wjcc.v12.i1.9 38292619 PMC10824181

[5] Quinn LM Wong FS Narendran P. Environmental determinants of type 1 diabetes: from association to proving causality. Front Immunol. (2021) 12:737964. 10.3389/fimmu.2021.737964 34659229 PMC8518604

[6] Felix KM Tahsin S Wu HJ. Host-microbiota interplay in mediating immune disorders. Ann NY Acad Sci. (2018) 1417:57–70. 10.1111/nyas.13508 28984367 PMC5889363

[7] Nogueira AR Shoenfeld Y. Microbiome and autoimmune diseases: cause and effect relationship. Curr Opin Rheumatol. (2019) 31:471–4. 10.1097/BOR.0000000000000628 31192811

[8] Vaarala O Atkinson MA Neu J. The "perfect storm" for type 1 diabetes: the complex interplay between intestinal microbiota, gut permeability, and mucosal immunity. Diabetes. (2008) 57:2555–62. 10.2337/db08-0331 18820210 PMC2551660

[9] Chan KL Tam TH Boroumand P Prescott D Costford SR Escalante NK Circulating NOD1 activators and hematopoietic NOD1 contribute to metabolic inflammation and insulin resistance. Cell Rep. (2017) 18:2415–26. 10.1016/j.celrep.2017.02.027 28273456

[10] Smith PM Howitt MR Panikov N Michaud M Gallini CA Bohlooly-Y M The microbial metabolites, short-chain fatty acids, regulate colonic treg cell homeostasis. Science. (2013) 341:569–73. 10.1126/science.1241165 23828891 PMC3807819

[11] Cardwell CR Stene LC Joner G Cinek O Svensson J Goldacre MJ Caesarean section is associated with an increased risk of childhood-onset type 1 diabetes mellitus: a meta-analysis of observational studies. Diabetologia. (2008) 51:726–35. 10.1007/s00125-008-0941-z 18292986

[12] Korpela K Salonen A Saxen H Nikkonen A Peltola V Jaakkola T Antibiotics in early life associate with specific gut microbiota signatures in a prospective longitudinal infant cohort. Pediatr Res. (2020) 88:438–43. 10.1038/s41390-020-0761-5 31954376

[13] Rampanelli E Nieuwdorp M. Gut microbiome in type 1 diabetes: the immunological perspective. Expert Rev Clin Immu. (2023) 19:93–109. 10.1080/1744666X.2023.2150612 36401835

[14] Hu S Kuwabara R de Haan BJ Smink AM de Vos P. Acetate and butyrate improve β-cell metabolism and mitochondrial respiration under oxidative stress. Int J Mol Sci. (2020) 21:1542. 10.3390/ijms21041542 32102422 PMC7073211

[15] Li Y Liu Y Chu CQ. Th17 cells in type 1 diabetes: role in the pathogenesis and regulation by gut microbiome. Mediat Inflamm. (2015) 2015:638470. 10.1155/2015/638470 PMC4710950 26843788

[16] Delzenne NM Cani PD. Gut microbiota and the pathogenesis of insulin resistance. Curr Diabetes Rep. (2011) 11:154–9. 10.1007/s11892-011-0191-1 21431853

[17] Aydin OC Aydın S Barun S. Role of natural products and intestinal flora on type 2 diabetes mellitus treatment. World J Clin Cases. (2023) 11:65–72. 10.12998/wjcc.v11.i1.65 36687192 PMC9846977

[18] Baroni I Fabrizi D Luciani M Magon A Conte G De Angeli G Probiotics and synbiotics for glycemic control in diabetes: a systematic review and meta-analysis of randomized controlled trials. Clin Nutr. (2024) 43:1041–61. 10.1016/j.clnu.2024.03.006 38527396

[19] Moravejolahkami AR Shakibaei M Fairley AM Sharma M. Probiotics, prebiotics, and synbiotics in type 1 diabetes mellitus: a systematic review and meta-analysis of clinical trials. Diabetes-Metab Res. (2024) 40:e3655. 10.1002/dmrr.3655 37183580

[20] Stefanaki C Rozou P Efthymiou V Xinias I Mastorakos G Bacopoulou F Impact of probiotics on the glycemic control of pediatric and adolescent individuals with type 1 diabetes: a systematic review and meta-analysis. Nutrients. (2024) 16:2629. 10.3390/nu16162629 39203766 PMC11357215

[21] Liberati A Altman DG Tetzlaff J Mulrow C Gøtzsche PC Ioannidis JP The PRISMA statement for reporting systematic reviews and meta-analyses of studies that evaluate health care interventions: explanation and elaboration. Ann Intern Med. (2009) 151:W65–94. 10.7326/0003-4819-151-4-200908180-00136 19622512

[22] Shi J Luo D Weng H Zeng X Lin L Chu H Optimally estimating the sample standard deviation from the five-number summary. Res Synth Methods. (2020) 11:641–54. 10.1002/jrsm.1429 32562361

[23] Luo D Wan X Liu J Tong T. Optimally estimating the sample mean from the sample size, median, mid-range, and/or mid-quartile range. Stat Methods Med Res. (2018) 27:1785–805. 10.1177/0962280216669183 27683581

[24] Huedo-Medina TB Sánchez-Meca J Marín-Martínez F Botella J. Assessing heterogeneity in meta-analysis: Q statistic or I^2^ index? Psychol Methods. (2006) 11:193–206. 10.1037/1082-989X.11.2.193 16784338

[25] Ho J Nicolucci AC Virtanen H Schick A Meddings J Reimer RA Effect of prebiotic on microbiota, intestinal permeability, and glycemic control in children with type 1 diabetes. J Clin Endocr Metab. (2019) 104:4427–40. 10.1210/jc.2019-00481 31188437

[26] Bianchini S Orabona C Camilloni B Berioli MG Argentiero A Matino D Effects of probiotic administration on immune responses of children and adolescents with type 1 diabetes to a quadrivalent inactivated influenza vaccine. Hum Vacc Immunother. (2020) 16:86–94. 10.1080/21645515.2019.1633877 PMC7012143 31210557

[27] Zare JA Aminzadeh M Haghighi-Zadeh MH Jamalvandi M. The effects of synbiotic supplementation on glycemic Status, lipid profile, and biomarkers of oxidative stress in type 1 diabetic patients. A placebo-controlled, double-blind, randomized clinical trial. Diabet Metab Synd Obes. (2020) 13:607–17. 10.2147/DMSO.S238867 PMC7060036 32184640

[28] Kumar S Kumar R Rohilla L Jacob N Yadav J Sachdeva N. A high potency multi-strain probiotic improves glycemic control in children with new-onset type 1 diabetes mellitus: a randomized, double-blind, and placebo-controlled pilot study. Pediatr Diabetes. (2021) 22:1014–22. 10.1111/pedi.13244 34174128

[29] Groele L Szajewska H Szalecki M Świderska J Wysocka-Mincewicz M Ochocińska A Lack of effect of lactobacillus rhamnosus GG and bifidobacterium lactis Bb12 on beta-cell function in children with newly diagnosed type 1 diabetes: a randomised controlled trial. BMJ Open Diab Res Care. (2021) 9:e001523. 10.1136/bmjdrc-2020-001523 33771763 PMC8006832

[30] Wang CH Yen HR Lu WL Ho HH Lin WY Kuo YW Adjuvant probiotics of lactobacillus salivarius subsp. salicinius AP-32, L. johnsonii MH-68, and Bifidobacterium animalis subsp. lactis CP-9 attenuate glycemic levels and inflammatory cytokines in patients with type 1 diabetes mellitus. Front Endocrinol. (2022) 13:754401. 10.3389/fendo.2022.754401 PMC8921459 35299968

[31] Shabani-Mirzaee H Haghshenas Z Malekiantaghi A Vigeh M Mahdavi F Eftekhari K. The effect of oral probiotics on glycated haemoglobin levels in children with type 1 diabetes mellitus—a randomized clinical trial. Pediatr Endocrinol Diabetes Metab. (2023) 29:128–33. 10.5114/pedm.2023.132025 38031828 PMC10679923

[32] Lokesh MN Kumar R Jacob N Sachdeva N Rawat A Yadav J Supplementation of high-strength oral probiotics improves immune regulation and preserves beta cells among children with new-onset type 1 diabetes mellitus: a randomised, double-blind placebo control trial. Indian J Pediatr. (2025) 92:277–83. 10.1007/s12098-024-05074-5 38557820

[33] Grahnemo L Nethander M Coward E Gabrielsen ME Sree S Billod JM Cross-sectional associations between the gut microbe Ruminococcus gnavus and features of the metabolic syndrome. Lancet Diabetes Endo. (2022) 10:481–3. 10.1016/S2213-8587(22)00113-9 35662399

[34] Nathan DM Buse JB Davidson MB Ferrannini E Holman RR Sherwin R Medical management of hyperglycemia in type 2 diabetes: a consensus algorithm for the initiation and adjustment of therapy: a consensus statement of the American diabetes association and the European association for the study of diabetes. Diabetes Care. (2009) 32:193–203. 10.2337/dc08-9025 18945920 PMC2606813

[35] Rittiphairoj T Pongpirul K Janchot K Mueller NT Li T. Probiotics contribute to glycemic control in patients with type 2 diabetes mellitus: a systematic review and meta-analysis. Adv Nutr. (2021) 12:722–34. 10.1093/advances/nmaa133 33126241 PMC8166562

[36] Wu R Luan J Hu J Li Z. Effect of probiotics on pregnancy outcomes in gestational diabetes: systematic review and meta-analysis. Arch Gynecol Obstet. (2024) 310:769–81. 10.1007/s00404-023-07346-5 38236281

[37] Çetinkaya ÖS Küçüktürkmen PB Metin T Dinçer B Sert H. The effect of probiotic and synbiotic use on glycemic control in women with gestational diabetes: a systematic review and meta-analysis. Diabetes Res Clin Pract. (2022) 194:110162. 10.1016/j.diabres.2022.110162 36403680

[38] Ayesha IE Monson NR Klair N Patel U Saxena A Patel D Probiotics and their role in the management of type 2 diabetes mellitus (short-term versus long-term effect): a systematic review and meta-analysis. Cureus J Med Sci. (2023) 15:e46741. 10.7759/cureus.46741 PMC10631563 38022046

[39] Wang X Chen L Zhang C Shi Q Zhu L Zhao S Effect of probiotics at different intervention time on glycemic control in patients with type 2 diabetes mellitus: a systematic review and meta-analysis. Front Endocrinol. (2024) 15:1392306. 10.3389/fendo.2024.1392306 PMC11303337 39114293

[40] Zarezadeh M Musazadeh V Faghfouri AH Sarmadi B Jamilian P Jamilian P Probiotic therapy, a novel and efficient adjuvant approach to improve glycemic status: an umbrella meta-analysis. Pharmacol Res. (2022) 183:106397. 10.1016/j.phrs.2022.106397 35981707

[41] Deng X Zheng C Wang S Yang R Liu Z Chen T. Treatment with a probiotic combination reduces abdominal adhesion in rats by decreasing intestinal inflammation and restoring microbial composition. Oncol Rep. (2020) 43:986–98. 10.3892/or.2020.7463 32020233

[42] Feng Y Wang Y Wang P Huang Y Wang F. Short-chain fatty acids manifest stimulative and protective effects on intestinal barrier function through the inhibition of NLRP3 inflammasome and autophagy. Cell Physiol Biochem. (2018) 49:190–205. 10.1159/000492853 30138914

[43] Yue X Wen S Long-Kun D Man Y Chang S Min Z Three important short-chain fatty acids (SCFAs) attenuate the inflammatory response induced by 5-FU and maintain the integrity of intestinal mucosal tight junction. BMC Immunol. (2022) 23:19. 10.1186/s12865-022-00495-3 35448938 PMC9027456

[44] Choi Y Choi SI Kim N Nam RH Jang JY Na HY Effect of clostridium butyricum on high-fat diet-induced intestinal inflammation and production of short-chain fatty acids. Digest Dis Sci. (2023) 68:2427–40. 10.1007/s10620-023-07835-2 36670324

[45] Jia L Shan K Pan LL Feng N Lv Z Sun Y Clostridium butyricum CGMCC0313.1 protects against autoimmune diabetes by modulating intestinal immune homeostasis and inducing pancreatic regulatory T cells. Front Immunol. (2017) 8:1345. 10.3389/fimmu.2017.01345 29097999 PMC5654235

[46] Huang S Li F Quan C Jin D. Intestinal flora: a potential pathogenesis mechanism and treatment strategy for type 1 diabetes mellitus. Gut Microbes. (2024) 16:2423024. 10.1080/19490976.2024.2423024 39520706 PMC11552262

[47] Greenhill C. Microbiota: FMT transiently improves insulin sensitivity. Nat Rev Endocrinol. (2017) 13:688. 10.1038/nrendo.2017.137 29027995

[48] Shen X Ma C Yang Y Liu X Wang B Wang Y The role and mechanism of probiotics supplementation in blood glucose regulation: a review. Foods. (2024) 13:2719. 10.3390/foods13172719 39272484 PMC11394447

[49] Lupien-Meilleur J Andrich DE Quinn S Micaelli-Baret C St-Amand R Roy D Interplay between gut microbiota and gastrointestinal peptides: potential outcomes on the regulation of glucose control. Can J Diabetes. (2020) 44:359–67. 10.1016/j.jcjd.2019.10.006 32057671

