# Supplementary material for: Effect of probiotics and related supplements on glycemic control in pediatric patients with type 1 diabetes mellitus: a systematic review and meta-analysis of clinical trials
Source: Front Pediatr. 2025 Oct 23;13:1633694. doi: 10.3389/fped.2025.1633694 (PMC12589916; doi:10.3389/fped.2025.1633694)
Supplement: Supplementary file 1 [file Table1.docx]

Supplementary Material

# Supplementary Figures and Tables

## Supplementary Figures

**
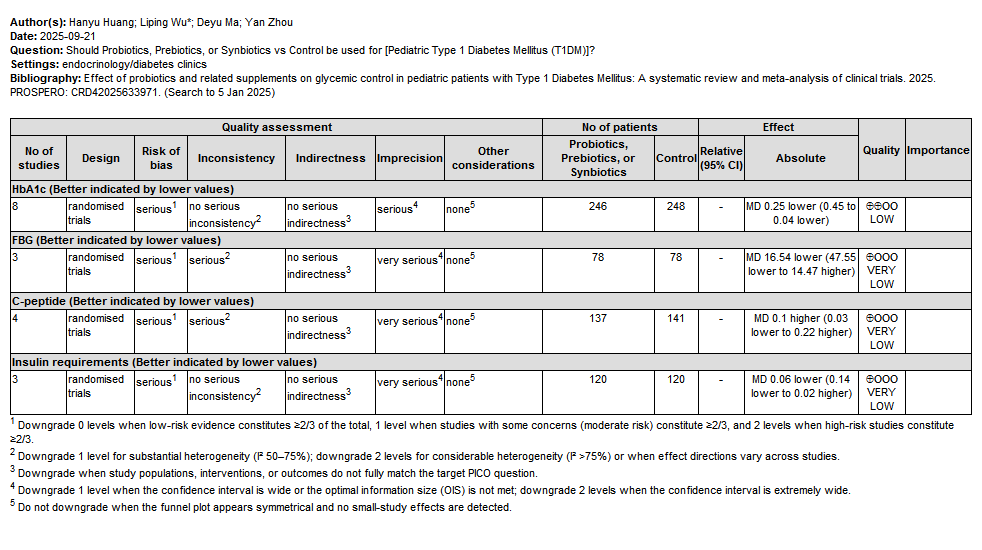
**

**Supplementary Figure 1.** GRADE Summary of Findings for Pediatric T1DM: Probiotics and Related Supplements vs Control (Glycemic Control).


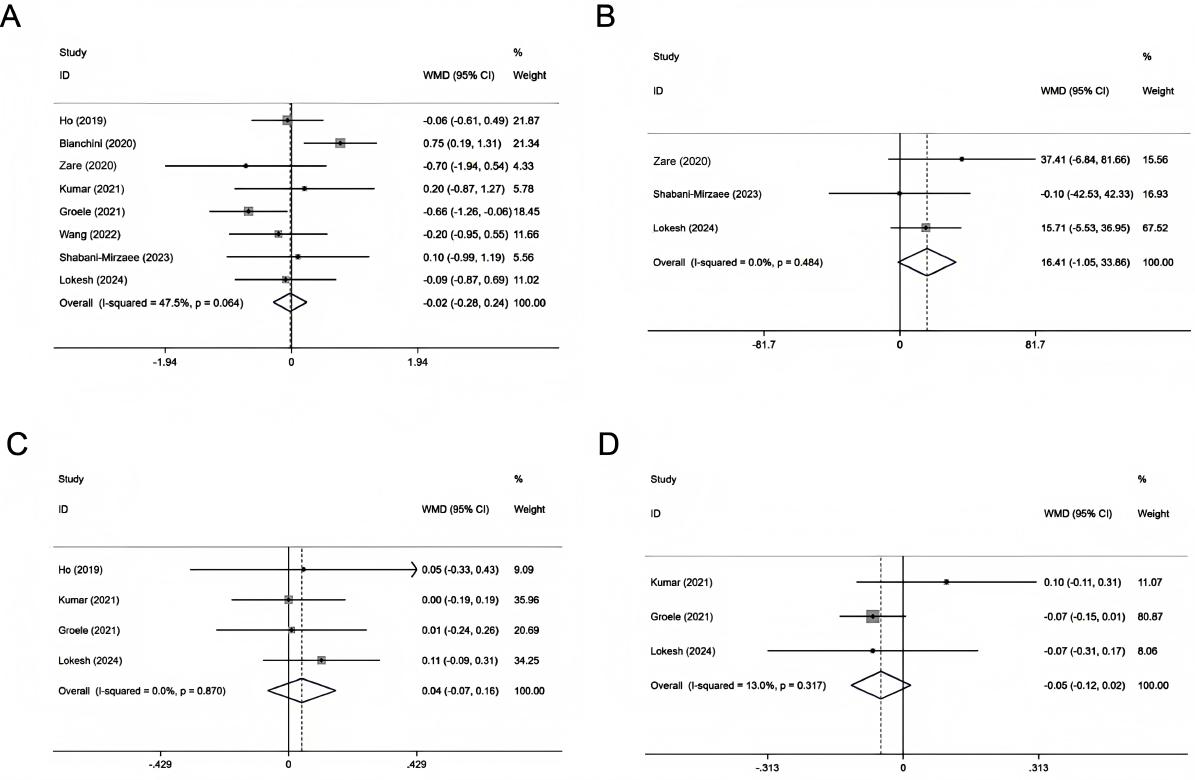


**Supplementary Figure 2.** The results of the baseline assessment, selected variables are HbA1c (A), FBG (B), C‐peptide (C), and insulin requirements (D).


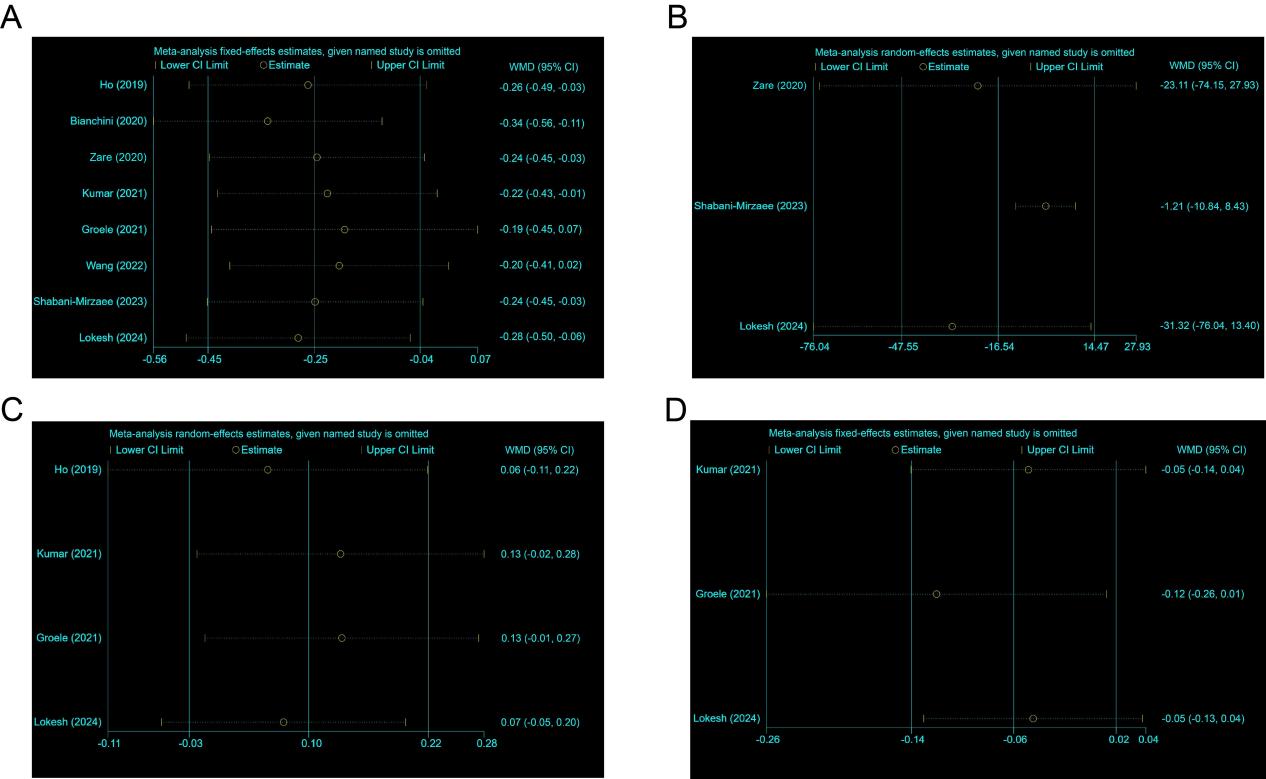


**Supplementary Figure 3.** Sensitivity Analysis of Meta-analysis Estimates (Study Omitted One-by-One), selected variables are HbA1c (A), FBG (B), C‐peptide (C), and insulin requirements (D).

**
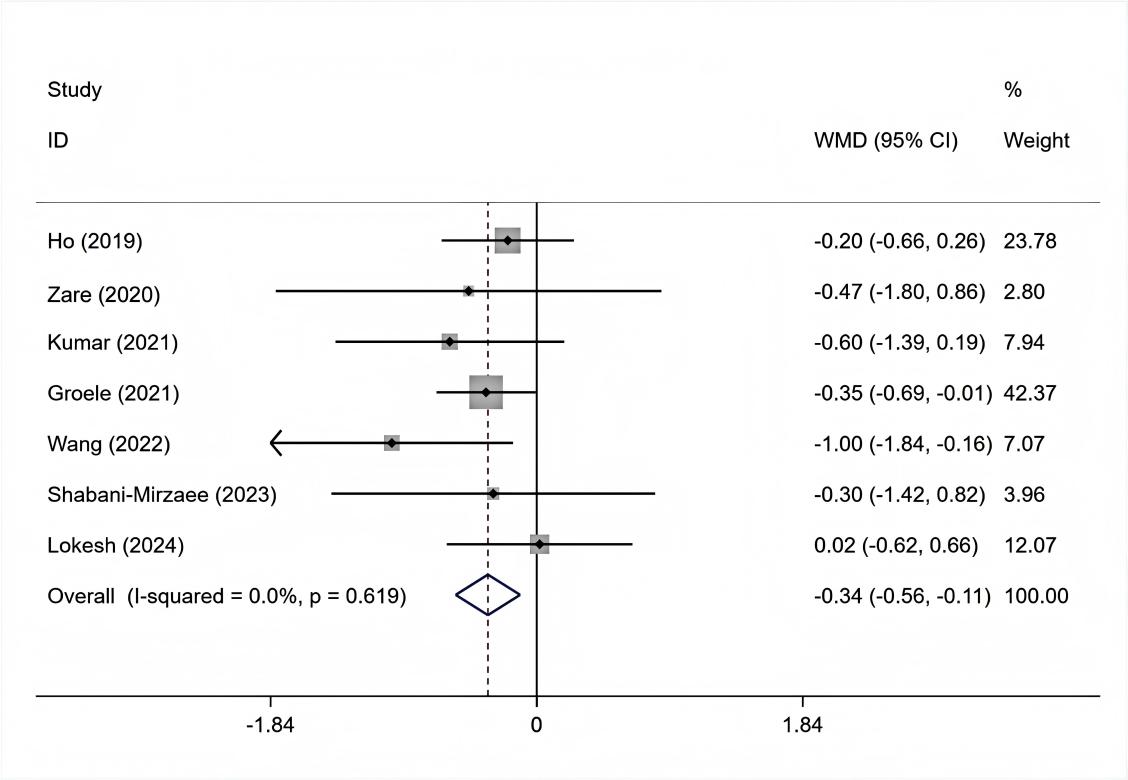
**

**Supplementary Figure 4.** Sensitivity Analysis Excluding the High Risk of Bias Study for the Outcome of HbA1c.


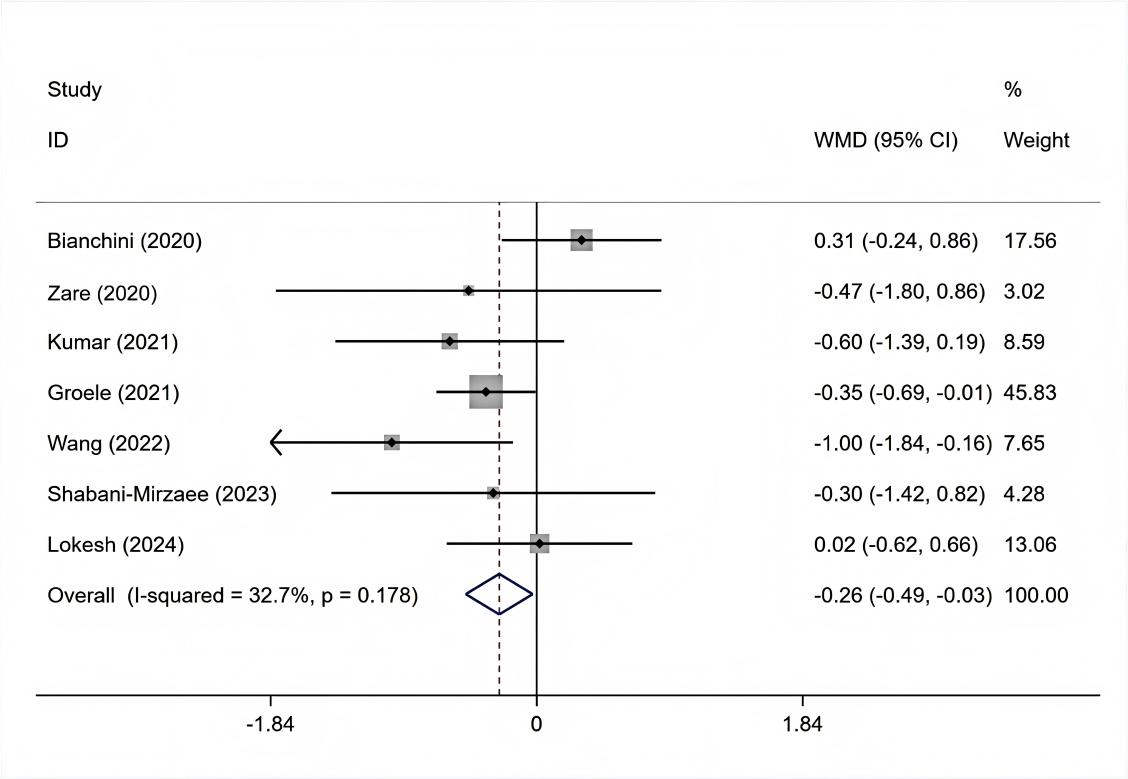


**Supplementary Figure 5.** Sensitivity Analysis Excluding the Prebiotic-Only Trial for the Outcome of HbA1c.

## Supplementary Tables

| Databases | Search terms |
| --- | --- |
| Pubmed  (n=29) | ((("Diabetes Mellitus, Type 1"[Mesh]) OR (((((((((((((((((((((((((((Type 1 Diabetes[Title/Abstract]) OR (Diabetes, Type 1[Title/Abstract])) OR (Diabetes Mellitus, Insulin-Dependent[Title/Abstract])) OR (Diabetes Mellitus, Insulin Dependent[Title/Abstract])) OR (Insulin-Dependent Diabetes Mellitus[Title/Abstract])) OR (Diabetes Mellitus, Juvenile-Onset[Title/Abstract])) OR (Diabetes Mellitus, Juvenile Onset[Title/Abstract])) OR (Juvenile-Onset Diabetes Mellitus[Title/Abstract])) OR (IDDM[Title/Abstract])) OR (Diabetes Mellitus, Type I[Title/Abstract])) OR (Diabetes Mellitus, Sudden-Onset[Title/Abstract])) OR (Diabetes Mellitus, Sudden Onset[Title/Abstract])) OR (Sudden-Onset Diabetes Mellitus[Title/Abstract])) OR (Type 1 Diabetes Mellitus[Title/Abstract])) OR (Diabetes Mellitus, Insulin-Dependent, 1[Title/Abstract])) OR (Insulin-Dependent Diabetes Mellitus 1[Title/Abstract])) OR (Insulin Dependent Diabetes Mellitus 1[Title/Abstract])) OR (Juvenile-Onset Diabetes[Title/Abstract])) OR (Diabetes, Juvenile-Onset[Title/Abstract])) OR (Juvenile Onset Diabetes[Title/Abstract])) OR (Diabetes, Autoimmune[Title/Abstract])) OR (Autoimmune Diabetes[Title/Abstract])) OR (Diabetes Mellitus, Brittle[Title/Abstract])) OR (Brittle Diabetes Mellitus[Title/Abstract])) OR (Diabetes Mellitus, Ketosis-Prone[Title/Abstract])) OR (Diabetes Mellitus, Ketosis Prone[Title/Abstract])) OR (Ketosis-Prone Diabetes Mellitus[Title/Abstract]))) AND (((("Probiotics"[Mesh]) OR (Probiotic[Title/Abstract])) OR (("Prebiotics"[Mesh]) OR (Prebiotic[Title/Abstract]))) OR (("Synbiotics"[Mesh]) OR (Synbiotic[Title/Abstract])))) AND (randomized controlled trial[Publication Type] OR randomized[Title/Abstract] OR placebo[Title/Abstract]) |

**Supplementary Table 1.** PubMed Search Strategy for Identifying RCTs on Probiotics in Type 1 Diabetes Mellitus.

| **Author/Year** | **Sample Size** | **Baseline HbA1c (%)** | **Post-intervention HbA1c (%)** | **Baseline FBG (mg/dl)** | **Post-intervention FBG (mg/dl)** | **Baseline C-peptide (ng/ml)** | **Post-intervention C-peptide (ng/ml)** | **Baseline Insulin Requirement (U/kg/d)** | **Post-intervention Insulin Requirement (U/kg/d)** |
| --- | --- | --- | --- | --- | --- | --- | --- | --- | --- |
| Ho 2019 | Intervention* | 8.02±0.82 | 7.94±0.5 | NA | NA | 0.26±0.75 | 0.31±0.09 | NA | NA |
|  | Control | 8.08±0.91 | 8.14±0.91 | NA | NA | 0.21±0.3 | 0.15±0.15 | NA | NA |
| Bianchini 2020 | Intervention | 8.45±1.29 | 8.24±1.25 | NA | NA | NA | NA | NA | NA |
|  | Control | 7.7±0.98 | 7.93±1 | NA | NA | NA | NA | NA | NA |
| Zare 2020 | Intervention | 8.90±1.95 | 8.61±1.85 | 199.72±81.10 | 163.68±75.88 | NA | NA | NA | NA |
|  | Control | 9.60±2.23 | 9.08±2.59 | 162.31±68.11 | 171.63±73.89 | NA | NA | NA | NA |
| Kumar 2021 | Intervention | 11.7±2.14 | 6.8±1.91 | 133.8±57.5 | NA | 0.3±0.38 | 0.5±0.46 | 1.0±0.54 | 0.6±0.31 |
|  | Control | 11.5±2.98 | 7.4±1.91 | 124.3±39.4 | NA | 0.3±0.53 | 0.5±0.38 | 0.9±0.46 | 0.7±0.46 |
| Groele 2021 | Intervention | 7.72±0.93 | 6.13±0.77 | NA | NA | 1.05±0.47 | 0.88±0.41 | 0.29±0.17 | 0.36±0.23 |
|  | Control | 8.38±1.83 | 6.48±0.88 | NA | NA | 1.04±0.72 | 0.89±0.46 | 0.36±0.2 | 0.39±0.25 |
| Wang 2022 | Intervention | 9.3±0.8 | 8.5±0.9 | NA | NA | NA | NA | NA | NA |
|  | Control | 9.5±1.9 | 9.5±2.1 | NA | NA | NA | NA | NA | NA |
| Shabani-Mirzaee 2023 | Intervention | 8.8±2.2 | 8.9±1.9 | 180.4±78.1 | 150.4±58.7 | NA | NA | NA | NA |
|  | Control | 8.7±1.8 | 9.2±2.2 | 180.5±78.0 | 204.0±92.3 | NA | NA | NA | NA |
| Lokesh 2024 | Intervention | 13.01±1.43 | 7.29±1.15 | 126±48.13 | 115.7±20.90 | 0.56±0.41 | 0.8±0.63 | 1.07±0.49 | 0.67±0.37 |
|  | Control | 13.1±1.63 | 7.27±1.37 | 110.29±34.75 | 116.57±18.01 | 0.45±0.36 | 0.45±0.76 | 1.14±0.47 | 0.84±0.51 |

**Supplementary Table 2.** Baseline and Post-intervention Outcomes in Included Studies. NOTES: Normally distributed quantitative variables are presented as mean ± SD. Abbreviations: NA, not available; HbA1c, hemoglobin A1c; C-peptide, fasting C-peptide; FBG, fasting blood glucose; *: Prebiotic, Probiotic, or Synbiotic.

| Study ID  (Authors and year) | Random sequence generation (selection bias) | Allocation concealment (selection bias) | Blinding of participants and personnel (performance bias) | Blinding of outcome assessment (detection bias) | Incomplete outcome data (attrition bias) | Selective reporting (reporting bias) | Other bias |
| --- | --- | --- | --- | --- | --- | --- | --- |
| Bianchini 2020 | Authors’ judgment :  Low risk | Authors’ judgment :  Low risk | Authors’ judgment :  Low risk | Authors’ judgment :  Unclear risk | Authors’ judgment :  high risk | Authors’ judgment :  Unclear risk | Low risk |
|  | Support for judgment :  Quote: "Starting from August 1,2017, selected patients were randomised 1:1" | Support for judgment :  Quote: "The study was conducted in blinded fashion by using identically labelled packaging for probiotic and placebo." | Support for judgment :  Quote:"physicians involved in clinical monitoring (SE, SB) were blinded to the treatment assignment." | Support for judgment :  The blinding of outcomes assessment was not mentioned. | Support for judgment :  The dropout rate is greater than 20%. | Support for judgment :  There is no information about this . |  |
| Groele 2021 | Authors’ judgment :  Low risk | Authors’ judgment :  Low risk | Authors’ judgment :  Low risk | Authors’ judgment :  Low risk | Authors’ judgment :  Low risk | Authors’ judgment :  Unclear risk | Low risk |
|  | Support for judgment :  Quote: “Block randomisation, with a block size of 4, was done with a computer-generated random number list prepared by an investigator with no clinical involvement in the trial. " | Support for judgment :  Quote: “The study products were packaged and assigned consecutive numbers according to the randomisation list by independent personnel." | Support for judgment :  Quote: "The list was concealed from the clinicians enrolling patients and assessing outcomes, as well as from the parents and the person responsible for the statistical analysis until completion of the study. " | Support for judgment :  Quote: "The list was concealed from the clinicians enrolling patients and assessing outcomes, as well as from the parents and the person responsible for the statistical analysis until completion of the study. " | Support for judgment :  Numbers of drop-outs and reasons were clearly described. | Support for judgment :  There is no information about this . |  |
| Ho 2019 | Authors’ judgment :  Low risk | Authors’ judgment :  Low risk | Authors’ judgment :  Low risk | Authors’ judgment :  Low risk | Authors’ judgment :  Low risk | Authors’ judgment :  Low risk | Low risk |
|  | Support for judgment :  Quote: "Participants were randomized 1:1 to the prebiotic or placebo group using computer-generated random numbers. " | Support for judgment :  Quote: "Both the patients and the research team were blinded to the group assignments. ". | Support for judgment :  Quote: "Both the patients and the research team were blinded to the group assignments. " | Support for judgment :  Quote: "Both the patients and the research team were blinded to the group assignments. " | Support for judgment :  Numbers of drop-outs and reasons were clearly described. | Support for judgment :  The measurements of outcomes described in the methods section were all reported. |  |
| Zare 2020 | Authors’ judgment :  Low risk | Authors’ judgment :  Low risk | Authors’ judgment :  Low risk | Authors’ judgment :  Unclear risk | Authors’ judgment :  Low risk | Authors’ judgment :  Unclear risk | Low risk |
|  | Support for judgment :  Quote: "Patients were randomly (block designbased on the combined analysis) divided into two intervention and control groups (25 subjects in each group)." | Support for judgment :  Quote: "Both the supplement and placebo powder were provided by the“Parsilact Company,” Shiraz, Iran. The placebo and supplement were matched in terms of shape, color, size, and taste." | Support for judgment :  Quote: "In this double-blind clinical trial, 50 T1DM patients were randomly allocated to intervention (n = 25) and control (n = 25) groups and received either synbiotic powder." | Support for judgment :  The blinding of outcomes assessment was not mentioned. | Support for judgment :  Numbers of drop-outs and reasons were clearly described. | Support for judgment :  There is no information about this . |  |
| Kumar 2021 | Authors’ judgment :  Low risk | Authors’ judgment :  Low risk | Authors’ judgment :  Low risk | Authors’ judgment :  Low risk | Authors’ judgment :  Low risk | Authors’ judgment :  Unclear risk | Low risk |
|  | Support for judgment :  Quote: " Computer-generated randomization using permuted blocks of 10 was prepared by an independent person, not involved in the study" | Support for judgment :  Quote: "The allocation sequence was concealed from the investigators, participants, and outcome assessors in sequentially numbered, opaque, sealed, and stapled envelopes." | Support for judgment :  Quote: " The master randomization list was blinded till the end of the study.The allocation sequence was concealed from the investigators, participants, and outcome assessors in sequentially numbered, opaque, sealed, and stapled envelopes" | Support for judgment :  Quote: " The master randomization list was blinded till the end of the study.The allocation sequence was concealed from the investigators, participants, and outcome assessors in sequentially numbered, opaque, sealed, and stapled envelopes" | Support for judgment :  Quote: " Both intention-to-treat (ITT) and per-protocol (PP) analysis were performed for main outcome variables." | Support for judgment :  There is no information about this . |  |
| Lokesh 2024 | Authors’ judgment :  Low risk | Authors’ judgment :  Low risk | Authors’ judgment :  Low risk | Authors’ judgment :  Unclear risk | Authors’ judgment :  Low risk | Authors’ judgment :  Unclear risk | Low risk |
|  | Support for judgment :  Quote:"All children fulfilling inclusion and exclusion criteria were randomised via a computer-generated algorithm into intervention (probiotic) and control (placebo) groups." | Support for judgment :  Quote:"The allocation concealment was done using opaque sealed envelopes. The same manufacturer provided both the probiotics and placebo as similar looking sachets for blinding." | Support for judgment :  Quote:"It was a randomised, double blinded, placebo-control trial" | Support for judgment :  The blinding of outcomes assessment was not mentioned. | Support for judgment :  Quote:"Intention-to-treat analysis was performed for the primary and secondary outcome parameters at the end of 6 mo intervention period. A P-value of less than 0.05 was considered statistically significant for all analysis. " | Support for judgment :  There is no information about this . |  |
| Shabani-Mirzaee 2023 | Authors’ judgment :  Low risk | Authors’ judgment :  Unclear risk | Authors’ judgment :  Low risk | Authors’ judgment :  Unclear risk | Authors’ judgment :  low risk | Authors’ judgment :  Unclear risk | Low risk |
|  | Support for judgment :  Quote:"To randomize the sampling, a computer number determinant was used." | Support for judgment :  The allocation concealment was not mentioned | Support for judgment :  Quote:"For single blinding of the study, participants in both groups (probiotic and control) were kept blind. " | Support for judgment :  The blinding of outcomes assessment was not mentioned. | Support for judgment :  All data of patient have been analyzed | Support for judgment :  There is no information about this . |  |
| Wang 2022 | Authors’ judgment :  Low risk | Authors’ judgment :  Unclear risk | Authors’ judgment :  Low risk | Authors’ judgment :  Unclear risk | Authors’ judgment :  Low risk | Authors’ judgment :  Unclear risk | Low risk |
|  | Support for judgment :  Quote:"Via computer-generated random numbering with double blinding, " | Support for judgment :  The allocation concealment was not mentioned | Support for judgment :  Quote:"Via computer-generated random numbering with double blinding, " | Support for judgment :  The blinding of outcomes assessment was not mentioned. | Support for judgment :  Numbers of drop-outs and reasons were clearly described. | Support for judgment :  There is no information about this . |  |

**Supplementary Table 3.** Risk of Bias Assessment for Randomized Controlled Trials According to the Cochrane Collaboration’s 'Risk of Bias' Tool.

| Subgroup | Heterogeneity | | Effect model | Meta analysis results | | |
| --- | --- | --- | --- | --- | --- | --- |
|  | I² | P |  | WMD | 95% CI (%) | P |
| ≤3 months | 5.5% | 0.375 | Fixed-effects model | -0.13 | (-0.43,0.17) | 0.405 |
| ＞3 months | 44.5% | 0.165 | Fixed-effects model | -0.35 | (-0.64,-0.07) | 0.015 |

**Supplementary Table 4.** Results of Subgroup Meta-analysis: Effect of Intervention Duration on HbA1c.

| Subgroup | Heterogeneity | | Effect model | Meta analysis results | | |
| --- | --- | --- | --- | --- | --- | --- |
|  | I² | P |  | WMD | 95% CI (%) | P |
| ≤2 months | 0.0% | 0.451 | Fixed-effects model | -0.31 | (-0.59,-0.03) | 0.031 |
| ＞2 months | 42.5% | 0.138 | Fixed-effects model | -0.17 | (-0.48,0.13) | 0.264 |

**Supplementary Table 5.** Results of Subgroup Meta-analysis: Effect of Disease Duration on HbA1c.

| Subgroup | Heterogeneity | | Effect model | Meta analysis results | | |
| --- | --- | --- | --- | --- | --- | --- |
|  | I² | P |  | WMD | 95% CI (%) | P |
| Single-strain ± Prebiotic | 11.3% | 0.288 | Fixed-effects model | 0.20 | (-0.31,0.71) | 0.452 |
| Multi-strain ± Prebiotic | 0.0% | 0.412 | Fixed-effects model | -0.38 | (-0.64,-0.12) | 0.005 |

**Supplementary Table 6.** Results of Subgroup Meta-analysis: Effect of Different Probiotics formulation on HbA1c.
